# Rate-distortion trade-off analysis of the power-domain ISAC waveform for the unmanned aerial vehicle

**DOI:** 10.1038/s41598-025-28895-6

**Published:** 2025-11-22

**Authors:** Gen Li, Lei Xiao, Junyi Du, Yanbo Sun, Dadong Ni, Yuansheng Wu, Jiale Wang, Yifan Guo, Jie Lian, Peng Wu

**Affiliations:** 1 Southwest China Institute of Electronic Technology, Chengdu, 610036 Sichuan China; 2 School of Marine Science and Technology, Northwestern Polytechnical University, Xi’an, 710072 Shaanxi China; 3 Xi’an Modern Control Technology Research Institute, Xi’an, 710072 Shaanxi China

**Keywords:** ISAC, UAV, OTFS, Power-domain, Rate, Distortion, Engineering, Physics

## Abstract

Integrated sensing and communication (ISAC) systems greatly promote the development of radio communication and sensing in the sixth-generation era. Meanwhile, the unmanned aerial vehicle (UAV) provides larger coverage and efficiency for multiple user communication and sensing due to the high mobility. Thus, the merging of these two techniques is promising for future wireless communication. However, the main problem is how to design a proper transmitting waveform that is suitable for high-mobility UAV communication while offering good sensing ability. In this paper, we propose the combination of the orthogonal time-frequency-space modulation (OTFS) and the frequency-modulated continuous wave (FMCW) using the power-domain superposition to achieve the high-mobility communication and sensing function. The OTFS modulation is used to resolve the Doppler shift of the communication and FMCW waveform guarantees the sensing ability. We also discuss the ratio-distortion trade-off between sensing and communication function. The numerical results validate the effectiveness of the proposed waveform.

## Introduction

Integrated sensing and communication (ISAC) systems embody a new trend in wireless technology, which brings the opportunity for wireless communication, such as wireless imaging^1,2^. These systems combine communication and sensing capabilities in a unified hardware framework to enhance resource utilization and cost-effectiveness^3–5^. Waveform design plays a crucial role in ISAC systems by creating waveforms that can efficiently support both sensing and communication functions at the same time^6–9^.

Based on the fundamental purpose of ISAC systems, the design techniques of ISAC waveforms are commonly grouped into communication-centric, sensing-centric, and joint waveforms. Furthermore, ISAC waveforms can be further classified as communication-based waveforms ^10–13^ and superposition waveforms^14–18^, according to structure of the waveforms. Communication-based ISAC waveforms represent that ISAC systems use standard communication signals, such as OFDM signals, or precoded communication signals as the transmitted signal to achieve the communication and sensing functions simultaneously. For standard communication waveforms, the ISAC systems generally do not pay additional attention to the communication function, however, it requires sophisticated estimation algorithms employed at the sensing receiver to extract target parameters from the communication echoes^10,11^. Although various parameter estimation algorithms are explored, the inherent randomness of communication signals still leads to terrible sensing performance^19^, failing to achieve the trade-off performance between communication and sensing of the ISAC systems. In order to achieve the trade-off performance, the researchers use precoding techniques to enhance the sensing ability of standard communication signals. They generally achieved a precoded ISAC waveform design by incorporating the beamforming matrix designs into the ISAC precoder, where the beamforming matrix is optimized considering the communication and sensing performance constraints simultaneously^12,13,20,21^.

Superposition waveforms combine random communication and specific sensing signals to form ISAC signals. They offer distinct advantages over communication-based waveforms. Notably, each ISAC symbol contains a deterministic sensing signal, ensuring reliable sensing performance per symbol rather than from a statistical viewpoint as seen in communication-based waveforms. Moreover, in ISAC systems that merge MIMO radar and multi-user communication, superposition waveforms allow the sensing function to maximize the virtual aperture^22^. Nevertheless, managing interference in superposition-waveform ISAC systems poses challenges. In an effort to enhance resource efficiency, some researchers explore the integration of sensing and communication within the same resource block, referred to as full-superposition waveforms. However, using this waveform makes it notably challenging to effectively mitigate sensing interference for communication signals. In^14^, authors propose a sensing interference-removing scheme inspired by NOMA and successive interference canceling (SIC) for full-superposition waveforms. The approach involves treating a portion of the sensing signals as virtual communication symbols and employing SIC to eliminate them, thereby reducing interference for actual communication symbols. However, the study overlooks the residual noise that remains post-removal of virtual communication symbols, which constitutes a portion of the sensing interference, in their theoretical framework. Moreover, in^23^, the authors incorporate part of the communication information into sensing signals, allowing users to enhance communication capacity through demodulation. Yet, they still fail to account for residual noise from the sensing signal post-SIC. So the communication performance is not satisfied. In order to reduce further the mutual-interference of sensing and communication signals, the authors introduced a quantization-based full-superposition waveform that presents a fresh approach for ISAC waveforms^15^, which involves quantizing the sensing signal solely at the ISAC transmitter. This technique has the potential to efficiently remove sensing interference at the communication receivers through a simpler operation compared to current methods.

The ISAC technique offers additional methods for communicating and monitoring specific objectives. Incorporating unmanned aerial vehicles (UAVs) into ISAC networks can significantly enhance their effectiveness. UAVs, being cost-effective aerial platforms, can improve ISAC services by providing broader coverage and a more reliable Line-of-Sight (LoS) connection. By capitalizing on UAVs’ mobility and strong air-to-ground LoS channels, UAV-enabled ISAC systems can deliver superior sensing and communication performance. However, this innovative approach presents unique design challenges, particularly related to the stringent size, weight, and power constraints imposed on UAVs, which can limit their communication and sensing capabilities. Therefore, formulating strategies for designing UAV-enabled ISAC systems to optimize sensing and communication performance while ensuring effective coordination among UAVs is a novel and intricate issue to tackle. Given the points above, there is a need to investigate a unified ISAC waveform for UAV-enabled systems to enhance spectrum efficiency. The superposition waveform emerges as a viable choice as it permits the reuse of power-domain resources, unencumbered by antenna aperture constraints, thereby reducing the size and payload requirements of UAVs.

**Fig. 1:**
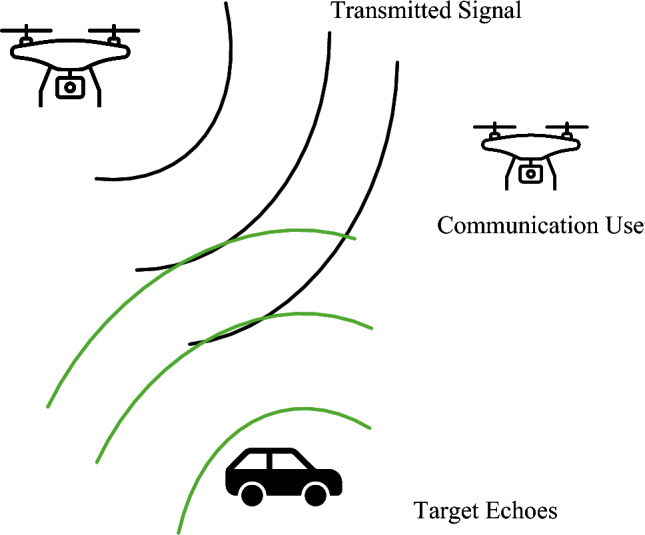
Illustration of the UAV-ISAC systems.

In this paper, we propose using the combination of orthogonal time-frequency-space modulation (OTFS) and power-domain superposition waveform to provide a robust communication sensing performance, as shown in Fig. 1. OTFS modulation maps information symbols into the delay–Doppler (DD) domain, allowing them to effectively capture time and frequency variations in high-mobility channels, which is crucial for UAV communication. Meanwhile, FMCW signals are classical radar waveforms that ensure high-precision range and velocity estimation, making them ideal for sensing tasks. Motivated by these properties, this paper combines OTFS and FMCW through a power-domain superposition scheme to achieve robust communication and sensing simultaneously. The power-domain superposition ISAC waveform is designed in the DD domain to provide sensing and communication abilities at the same time-frequency-space resource. Then, the ISAC waveform is modulated by OTFS in order to fit in the high-mobility communication channel due to the UAV flying. We also discuss the optimal power-domain ISAC waveform in terms of the rate-distortion trade-off viewpoint, where the power allocation index and quantization order are optimized according to the sensing and communication requirements. The simulation results demonstrate the effectiveness of the proposed waveform for UAV communication and sensing.

## Transmitted signal model

In this paper, we consider a UAV with sensing and communication functions using the same time-frequency resources. For UAVs, they generally are controlled to form a special swarm for some specific tasks, so they need to communicate and sense with each other to keep the formation. Now, we first introduce the transmitted ISAC signal and received signals at the communication end and sensing end model, respectively. Notably, we consider the mono-static ISAC system in this work where the sensing receiver and ISAC transmitter are colocated.

### Power-domain ISAC waveform

Due to the high mobility property of the UAV, the Doppler frequency shift is severe, degrading the performance of conventional communication schemes, such as orthogonal frequency division multiplexing (OFDM). In order to remove the Doppler frequency shift easily, the OTFS modulation is used.

At first, we generate a binary sequence. Then, the binary sequence is mapped into a symbol using a *Q*-QAM constellation. After that, the symbols are formed as an $M\times N$ matrix $\textbf{X}_c$

1 $$\begin{aligned} \textbf{X}_c = \left[ \begin{aligned} x^c_{0,0} ~&x^c_{0,1}~&\cdots ~&x^c_{0,M-1} \\ x^c_{1,0} ~&x^c_{1,1}~&\cdots ~&x^c_{1,M-1} \\ \vdots ~&\vdots ~&\cdots ~&\vdots \\ x^c_{N-1,0} ~&x^c_{N-1,1}~&\cdots ~&x^c_{N-1,M-1} \end{aligned} \right] , \end{aligned}$$

where *M* and *N* denote the numbers of delay bins and Doppler bins in the DD domain, respectively. The matrix $\textbf{X}_c$ represents the conveyed communication information in the Delay-Doppler domain. Then, we introduce the design of the sensing signal in the DD domain, and the two signals are combined to generate the power-domain ISAC signal.

As stated in Ref.^15^, the power-domain ISAC waveform design relies on the quantization of the sensing signal. Thus, we generate the sensing radar signal. To align with existing radar systems, we use the classical frequency-modulated continuous wave (FMCW) signal as the original radar signal, which is generated by

2 $$\begin{aligned} \begin{aligned} f(t)&= \sum _{m=0}^{M-1}e^{j2\pi (f_0t+\kappa t^2)}G_{\textrm{RC}}(t - mT)\\&= \sum _{m=0}^{M-1}e^{j2\pi (f_0t+B/T t^2)}G_{\textrm{RC}}(t - mT), \end{aligned} \end{aligned}$$

where *j* is the imaginary unit, $f_0$ is the carrier frequency, and *M* is the number of the linear frequency-modulated (LFM) signal. $\kappa = B/T$ is the frequency slope, where *B* is the bandwidth of the FMCW signal, *T* is the duration of the LFM signal, and

3 $$\begin{aligned} G_{\textrm{RC}}(t)= {\left\{ \begin{array}{ll} \dfrac{1}{T}\!\left[ 1+\cos \!\left( \dfrac{\pi t}{\beta T}\right) \right] , & |t|\le \dfrac{\beta T}{2}, \\[8pt] \dfrac{1}{T}, & \dfrac{\beta T}{2}<|t|\le \dfrac{T}{2}, \\[8pt] 0, & \text {otherwise}, \end{array}\right. } \end{aligned}$$

is a time-limited function, where $\beta \in [0,1]$ denotes the roll-off factor that controls the smoothness of the pulse edges. The use of $G_{\textrm{RC}}(t)$ provides a smooth transition region, which reduces spectral sidelobes and inter-symbol interference. Notably, we need to generate the power-domain ISAC signal in the baseband, so the FMCW signal used here is also generated in the baseband.

Due to the use of the OTFS modulation, we should process the signal in the DD domain. Therefore, the original radar signal should also be converted into the DD domain for further processing. Now, we sample the *m*-th LFM of the FMCW signal in (2) with a sampling rate *B* as

4 $$\begin{aligned} {s}_m(n) = e^{j2\pi \kappa n /B^2}, n = 0,\cdots ,N-1, \end{aligned}$$

where $\boldsymbol{s}$ is the $N\times 1$ vector, representing the discrete version of the LFM signal. Then, the FMCW signal could be denoted as

5 $$\begin{aligned} f(n) = \sum _{m=0}^{M}{s}_m (n-m(N-1)) . \end{aligned}$$

Then the delay-time domain signal *f*(*n*) is transformed to the time-frequency domain using the Wigner transform to obtain $\textbf{f}$. The symplectic fast Fourier Transform (SFFT) is applied to the time-frequency domain matrix $\textbf{f}$ to obtain the delay-Doppler domain form

6 $$\begin{aligned} F_{n,m} = \frac{1}{\sqrt{MN}}\sum _{k=0}^{N-1}\sum _{l=0}^{M-1}\text {f}[l,k] e^{-j2\pi (\frac{nk}{N}-\frac{ml}{M})}, \end{aligned}$$

where $\text {f}[l,k]$ is the *l*th row, *k*th column element of $\textbf{f}$. Then the $F_{n,m}$ could also form a matrix as

7 $$\begin{aligned} \textbf{F} = \left[ \begin{aligned} F_{0,0} ~&F_{0,1}~&\cdots ~&F_{0,M-1} \\ F_{1,0} ~&F_{1,1}~&\cdots ~&F_{1,M-1} \\ \vdots ~&\vdots ~&\cdots ~&\vdots \\ F_{N-1,0} ~&F_{N-1,1}~&\cdots ~&F_{N-1,M-1} \end{aligned} \right] . \end{aligned}$$

Notably, because of the property of the DD domain for this periodic signal *f*(*t*), the elements of the matrix $\textbf{F}$ satisfy

8 $$\begin{aligned} F_{n,m}|_{n\ne 0} = 0. \end{aligned}$$

In other words, the element of the first column of $\textbf{F}$ is non-zero, but the other elements must be zero. This property will help in signal separation at the communication user, providing an advantage, as we will show in the next section.

Then, we should quantize the sensing signal matrix $\textbf{F}$ to enable the power-domain ISAC waveform as

9 $$\begin{aligned} \textbf{X}^s = Q(\textbf{F}, L_s). \end{aligned}$$

The operator $Q(\cdot , L_s)$ represents a uniform real-valued quantization function with quantization order $L_s$ , applied to the sensing signal matrix $\textbf{F}$ to obtain the quantized sensing waveform $\textbf{X}^s$. In this paper, the quantizer has the following requirements: (1) The order $L_s$ should be odd due to some elements of $\textbf{F}$ being zero; (2) the uniform quantizer is considered for simplicity; (3) The real-valued quantizer is used because the $\textbf{F}$ is real-valued. For the general sensing signal, the complex-valued quantization could be considered. According to these requirements, the constellation of quantized sensing signals could be written as

10 $$\begin{aligned} {X}^s_{n,m} \in \left\{ - {X}^{+s}, {X}^{+s}\right\} , \end{aligned}$$

where

11 $$\begin{aligned} {X}^{+s} \in \left\{ \max \{|\textbf{F}|\},~\cdots ,~\max \{|\textbf{F}|\}-l\frac{2\max \{|\textbf{F}|\}}{L_s-1},~\cdots ,~ 0 \right\} . \end{aligned}$$

After obtaining the communication and sensing signals, we utilize the two signals to generate the ISAC waveform in DD domain. The power-domain ISAC waveform is

12 $$\begin{aligned} \textbf{X} = \sqrt{P_s}\textbf{X}^s/\sigma _s^2 + \sqrt{P_c}\textbf{X}^c/\sigma _c^2, \end{aligned}$$

where $\sigma _s^2$ and $\sigma _c^2$ are the power of signal $\textbf{X}^s$ and $\textbf{X}^c$, respectively. $P_s$ and $P_c$ are the allocated power for sensing and communication signals, with $P_s + P_c = P_t$, where $P_t$ is the total transmitting power. Additionally, $P_s \ge P_c$ because the radar signal generally involves round-trip propagation in the studied monostatic scenario, and the radar cross-section (RCS) of the target is considered weak. Under this scenario, $P_s \ge P_c$ is reasonable.

Then the DD domain ISAC signal is converted into the time-frequency domain to be emitted by using

13 $$\begin{aligned} \text {x}[k,l] = \sqrt{MN}\sum _{n=0}^{N-1}\sum _{m=0}^{M-1}X[n,m] e^{j2\pi (\frac{nk}{N}-\frac{ml}{M})}, \end{aligned}$$

where *X*[*n*, *m*] is the *n*th row and *m*th column of the matrix $\textbf{X}$. Finally, we use the Heisenberg transform and add the cyclic prefix (CP) in order to obtain the time-domain transmitting signal *x*(*t*). The signal *x*(*t*) is then converted and emitted from an antenna on the UAVs.

## Received signal model

In this section, we describe the received signal models at the communication user and sensing receiver.

### Received communication signal

For the high mobility communication user, the Doppler effect is significant, so the communication channel between transmitter and communication receiver with delay-Doppler representation is

14 $$\begin{aligned} h(\tau ,\mu ) = \sum _{k=1}^{K}g_k\delta (\tau -\tau _k)\delta (\mu -\mu _k), \end{aligned}$$

where $\tau$, $\mu$ are the delay and Doppler frequency shift, and *K* is the number of the propagation paths. $g_k$ is the complex channel gain, and $\delta$ is the Dirac function. Then the received communication signal passing the channel, after down-convert, could be written as

15 $$\begin{aligned} r(t) = \int \int h(\tau ,\mu )e^{j2\pi \mu t}x(t-\tau ) d \mu d \tau +n_c(t). \end{aligned}$$

where $n_c(t)$ is the zero-mean additive Gaussian white noise with variance $\sigma ^2_n$. When the CP is removed, the signal is converted from serial to parallel, and the Wigner transform and inverse SFFT are executed on it to convert the received signal into the DD domain. The signal in the DD domain is

16 $$\begin{aligned} \textbf{R} = \textbf{H} *\textbf{X} + \textbf{N}_c, \end{aligned}$$

where $\textbf{H}$ represents the sampled matrix form of the channel $h(\tau ,\mu )$, $*$ denotes the two-dimensional circular convolution operation, and $\textbf{N}_c$ is the DD domain noise of $n_c(t)$. In accordance with Reference ^24^, the linear minimum mean square error (LMMSE) equalizer is utilized to remove the channel. Define the vectorized forms $\textbf{r}=\textrm{vec}(\textbf{R})$, $\textbf{x}=\textrm{vec}(\textbf{X})$, and $\textbf{n}_c=\textrm{vec}(\textbf{N}_c)$, where $\textrm{vec}(\cdot )$ is the vectorized operation. Then the system can be written in vector form as

17 $$\begin{aligned} \textbf{r} = C(\textbf{H})\textbf{x} + \textbf{n}_c, \end{aligned}$$

where $C(\textbf{H})\in \mathbb {C}^{NM\times NM}$ is the 2-dimension circular convolution matrix, constructed as

18 $$\begin{aligned} C(\textbf{H}) = \sum _{n=0}^{N-1}\sum _{m=0}^{M-1} \textbf{H}[n,m]\,\big (\textbf{P}_N^{n}*\textbf{P}_M^{m}\big ), \end{aligned}$$

with $\textbf{P}_N$ and $\textbf{P}_M$ being the $N\times N$ and $M\times M$ cyclic permutation matrices. The LMMSE equalizer minimizing $\mathbb {E}\{\Vert \textbf{x} - \textbf{G}_{MMSE} \textbf{r}\Vert ^2\}$ is

19 $$\begin{aligned} \textbf{G}_{MMSE} = \boldsymbol{\Sigma _x}C(\textbf{H})^{H}\!\big (C(\textbf{H})\,\boldsymbol{\Sigma _x}C(\textbf{H})^{H} + \boldsymbol{\Sigma _n}\big )^{-1}, \end{aligned}$$

where $\boldsymbol{\Sigma _x}=\mathbb {E}[\textbf{x} \textbf{x}^{H}]$ and $\boldsymbol{\Sigma _n}=\mathbb {E}[\textbf{n}_c \textbf{n}_c^{H}]$. For independent and identically distributed transmitted symbols and white Gaussian noise, $\boldsymbol{\Sigma _x}=\sigma _x^2 \textbf{I}$, $\boldsymbol{\Sigma _n}=\sigma _n^2 \textbf{I}$, giving the LMMSE equalizer as

20 $$\begin{aligned} \textbf{G}_{MMSE} = \big (C(\textbf{H})^{H}C(\textbf{H}) + \tfrac{\sigma _n^2}{\sigma _x^2}\textbf{I}\big )^{-1}C(\textbf{H})^{H}. \end{aligned}$$

After equalizing the channel, (16) is processed as

21 $$\begin{aligned} \hat{\textbf{X}} = \sqrt{P_s}\textbf{X}^s/\sigma _s^2 + \sqrt{P_c}\textbf{X}^c/\sigma _c^2+\textbf{N}_{c,eq}. \end{aligned}$$

For the communication receiver, the vital problem is to extract the communication symbol $\textbf{X}^c$ from $\hat{\textbf{X}}$. In order to obtain the communication signal, the quantizer is used again as stated in (9). However, the DD domain has a unique property for this operation. Back to (8), the elements of $\textbf{F}$ are zero for the case $n\ne 0$, leading to the elements of $\textbf{X}^s$ also being zero. Thus, we only need to process the first column of $\hat{\textbf{X}}$, i.e., $\hat{\textbf{x}}_1$, to obtain the quantized sensing symbol $\textbf{X}^s$. We denote the first column of ${\textbf{X}}^s$ as ${\textbf{x}}^s$, the first column of ${\textbf{X}}^c$ as ${\textbf{x}}^c$. Notably, the power ratio between ${\textbf{x}}^s$ and ${\textbf{x}}^c$ is $(M-1)P_s/P_c$, the gain $M-1$ comes from the zero elements of $\textbf{X}^s$. Because the $P_s\ge P_c$, the communication symbol ${\textbf{x}}^c$ could be viewed as a weaker interference for the quantized sensing symbol ${\textbf{x}}^c$. Therefore, we could obtain

22 $$\begin{aligned} \hat{{\textbf{x}}}^s = Q(\hat{\textbf{x}}_1/\sqrt{P_s}, L_s). \end{aligned}$$

Then, the estimation of ${{\textbf{X}}}^s$, i.e.,$\hat{{\textbf{X}}}^s$, could be obtained by using $\hat{{\textbf{x}}}^s$ and zero padding.

Now, the communication symbols could be obtained as

23 $$\begin{aligned} \begin{aligned} \hat{{\textbf{X}}}^c&= \sigma _c^2 \frac{\hat{\textbf{X}}-\sqrt{P_s}\hat{\textbf{X}}^s/\sigma _s^2}{\sqrt{P_c}} \\&= \sigma _c^2 \frac{ \sqrt{P_s}(\textbf{X}^s-\hat{\textbf{X}}^s)/\sigma _s^2 + \sqrt{P_c}\textbf{X}^c/\sigma _c^2+\textbf{N}_{c,eq}}{\sqrt{P_c}} \\&\approx \textbf{X}^c+ \sigma _c^2 \frac{\textbf{N}_{c,eq}}{\sqrt{P_c}}, \end{aligned} \end{aligned}$$

where the approximation is valid when $P_s\ge P_c$. This approximation is employed solely to illustrate the estimated communication symbols when the sensing symbol is assumed to be perfectly estimated. In the simulation section, we refrain from utilizing this approximation and instead opt for the actual demodulation performance.

From the processing flow, we see that the combination of the FMCW signal and OTFS modulation is more suitable for the power-domain ISAC waveform than OFDM in^15^. The $M-1$ times gain is obtained through the mean operation across multiple OFDM symbols, leading to additional operations and increased complexity. This is also the main advantage of the proposed method.

## Rate-distortion trade-off waveform optimization

In the previous section, we introduced the combination of OTFS modulation and FMCW signal for ISAC. However, two vital parameters $L_s$ and $P_s (P_c)$ impact the sensing and communication performance significantly. Some metrics are proposed to evaluate the communication and sensing performance in ISAC systems, such as mutual information^25^ and waveform similarity^26^. In this section, we will discuss the optimal parameter selection based on the rate-distortion trade-off for different performance requirements.

### Sensing distortion

For the sensing function, the transmitted waveform is important because it determines the lower bound of the sensing performance. However, the performance metrics of the sensing waveform are various, such as Cramér-Rao bound, peak-to-average power ratio, and constant modulus. Therefore, justifying that an ISAC waveform has a better sensing performance is a challenging task.

A reasonable way is to measure the difference between the ISAC waveform and the classical radar waveform, such as the FMCW signal. The classical radar waveforms are generally widely used and have robust sensing performance, so it could be viewed as a reference waveform for sensing function. In this paper, we use the Euclidean distance between the ISAC waveform and the FMCW signal because our ISAC waveform is the combination of communication signal and FMCW. And we named this distance as waveform distortion

24 $$\begin{aligned} \begin{aligned} D(L_s,P_s)&= ||\textbf{X}- \frac{\sqrt{P_t}}{\sigma _s^2}\textbf{F}||_2^2\\&= ||\sqrt{P_s}\textbf{X}^s/\sigma _s^2 + \sqrt{P_c}\textbf{X}^c/\sigma _c^2- {\sqrt{P_t}}\textbf{F}/{\sigma _s^2}||_2^2\\&=||\sqrt{P_s}Q(\textbf{F},L_s)/\sigma _s^2 + \sqrt{P_t-P_s}\textbf{X}^c/\sigma _c^2- {\sqrt{P_t}}\textbf{F}/{\sigma _s^2}||_2^2. \end{aligned} \end{aligned}$$

From (24), we see that the distortion of the sensing signal is impacted by the parameters $L_s$ and $P_s$. Meanwhile, when $P_s$ approaches $P_t$, the second term of the third line in (24) is nearly zero. The distortion is only related to the quantization order, and the lower the quantization order, the higher the distortion. When the quantization order $L_s$ approaches infinity, i.e., without quantization, the distortion $D(L_s, P_s)$ becomes zero. In other words, the sensing performance of the ISAC waveform and FMCW signal is the same. Thus, we desire the distortion $D(L_s, P_s)$ to be as low as possible for better sensing performance in the ISAC systems.

### Communication rate

For the communication systems, the communication achievable rate is an important metric, which represents the ability of the conveying information to the communication systems. In the proposed system, we could obtain the estimation of $\textbf{X}^c$ using (23), thus the communication capacity, i.e., maximum rate, is

25 $$\begin{aligned} \begin{aligned} C_1(P_s,L_s)&= B\log _2\left( 1+\frac{\sigma _c^2}{ {P_s}/P_c||\textbf{X}^s-\hat{\textbf{X}}^s||_2^2 \sigma _c^2/\sigma _s^2 +\sigma _c^2\sigma _n^2/P_c } \right) \\&\approx B\log _2\left( 1+\frac{1}{\sigma _n^2/P_c } \right) , \end{aligned} \end{aligned}$$

where the approximation also is based on the perfect demodulation of the quantized sensing symbol, as shown in (22). However, this capacity has two limitations for the proposed system. First, (25) is valid if the input $\textbf{X}^c$ is subject to a Gaussian distribution, but the real communication symbols $\textbf{X}^c$ in our paper are extracted from an alphabet, leading to the ineffectiveness of this equation. Second, this approximation is ideal when we analyze the impact of the parameters on the performance of this ISAC system, which is held when the $P_s$ is larger than $P_c$. According to these approximations, we could not see the whole trade-off property in terms of the adjustable parameters. We need to derive the more proper form of the capacity for this system.

At first, the communication rate could be calculated as

26 $$\begin{aligned} R(P_s,L_s) = I(\textbf{X}^c)-I(\textbf{X}^c| \sqrt{P_s},\textbf{X}^s-\hat{\textbf{X}}^s,L_s), \end{aligned}$$

where the *I* denotes the entropy of the information. The capacity under the used system is

27 $$\begin{aligned} C(P_s,L_s) = \max _{P_s,L_s}R(P_s,L_s). \end{aligned}$$

According to the definition of entropy of the discrete symbols, we have

28 $$\begin{aligned} I(\textbf{X}^c) = -\sum _{i_1}^{U_1}P(x^c_{i_1})\log _2P(x^c_{i_1}), \end{aligned}$$

and

29 $$\begin{aligned} I(\textbf{X}^c| \sqrt{P_s},\textbf{X}^s-\hat{\textbf{X}}^s,L_s) = -\int P(y)\sum _{i_1}^{U_1} \left[ P(x_{i_1}^c|y)\log _2P(x_{i_1}^c|y) \right] dy , \end{aligned}$$

where

30 $$\begin{aligned} y= \sigma _c^2 \frac{ \sqrt{P_s}({x}^s_{i_1} -\hat{{x}}^s_{i_1} )/\sigma _s^2 + \sqrt{P_c}{x}^c_{i_2} /\sigma _c^2+{n}_{c,eq}}{\sqrt{P_c}}. \end{aligned}$$

Equation (29) shows that the mutual information $I(\textbf{X}^c| \sqrt{P_s},\textbf{X}^s-\hat{\textbf{X}}^s,L_s)$ is related with the parameters $P_s$, constellation of transmitted symbols and the noise level. In fact, the quantization order $L_s$ is implicitly contained in the expression due to the term $\textbf{X}^s-\hat{\textbf{X}}^s$. The quantization order $L_s$ and power index $P_s$ impact the value of $\textbf{X}^s-\hat{\textbf{X}}^s$.

However, Eq. (29) could not be calculated analytically, because the statistical property of the *y* is complex and unknown to us. Therefore, we need to obtain it using the Monte Carlo simulation. According to Ref.^27^, we have

31 $$\begin{aligned} R(P_s,L_s) = \log _2 M_c - \frac{1}{M_c}\sum _{x^c \in \mathscr {X}^c}\mathbb {E}[A(y,x^c)|x^c], \end{aligned}$$

where $M_c$ is the modulation size of the communication symbols,

32 $$\begin{aligned} A(y,x^c) = \log _2 \frac{ \sum _{x^{c} \in \mathscr {X}^c}\sum _{x_1 \in \mathscr {X}_1} e^{-\frac{P_c|y-(x_1+x^{c} )|^2}{2\sigma _n^2}} }{ \sum _{x_1 \in \mathscr {X}_1} e^{-\frac{P_c|y-(x_1+x^{c} )|^2}{2\sigma _n^2}} } \end{aligned}$$

and

33 $$\begin{aligned} x_1 = \sigma _c^2 /\sigma _s^2 \sqrt{P_s}/\sqrt{P_c}({x}^s_{i_1} -\hat{{x}}^s_{i_1} ). \end{aligned}$$

The variable *y* is subjected to a random distribution, and it probability density function is

34 $$\begin{aligned} p(y|x^c,x_1) = \frac{1}{\sqrt{2\pi \sigma _n^2}}e^{-\frac{|y-(x_1+x^{c} )|^2}{2\sigma _n^2}} . \end{aligned}$$

We could obtain the $R(L_s,P_s)$ by using Monte-Carlo simulation through (31) to (34).

### Trade-off waveform

In the previous subsections, we introduced the sensing distortion and communication rate of the proposed power-domain ISAC waveform. Now, we will discuss the influence of the two adjustable parameters $P_s$ and $L_s$ on these two metrics. For the sensing distortion $D(L_s, P_s)$, we see that the larger $P_s$ is, the larger $L_s$ is, and the better (smaller) the sensing distortion is. In contrast, the larger $P_s$ is, the larger $L_s$ is, and the worse the communication rate is. Therefore, the sensing distortion and communication rate conflict in terms of the two adjustable parameters, as described in common ISAC systems. In order to depict the whole trade-off property of the used power-domain waveform, the following problem should be studied:

35 $$\begin{aligned} \begin{aligned}&\min _{P_s,P_c,L_s} D(L_s, P_s)\\ s.t. \quad&R(P_s,L_s)\ge R_0\\&P_c+P_s = P_t. \end{aligned} \end{aligned}$$

We change the value of $R_0$ to obtain different values of $P_s$ and $L_s$ in order to reveal the trade-off property between sensing distortion and communication rate using the MCS.

## Results

In this section, we examine the proposed ISAC waveform aimed for the UAV, focusing on the communication and sensing performance. Finally, we demonstrate the trade-off property in terms of the rate-distortion criterion. We assume that a UAV is communicating with other users and sensing the environment. The simulation parameters are listed in Table 1.

**Table 1:** The simulation parameters.

Parameter	Value
Maximum Velocity of UAV	700km/h
N	64
M	16
$P_t$	1
$\sigma _n^2$	0.01
$M_c$	4
Carrier Frequency	5.8 GHz
Frequency Spacing $\Delta f$	12.5 KHz

### Communication performance

First, we show the communication performance. Considering the line-of-sight (LoS) path of the UAVs, we only assume that the communication user receives the one path with the Doppler shift and delay, and the equalizer is used to remove the channel effect. Notably, the OTFS modulation is used in our paper, so the communication system has the ability to combat the Doppler shift. The communication channel is randomly generated in each simulation, and the results shown here are the statistic property.

**Fig. 2:**
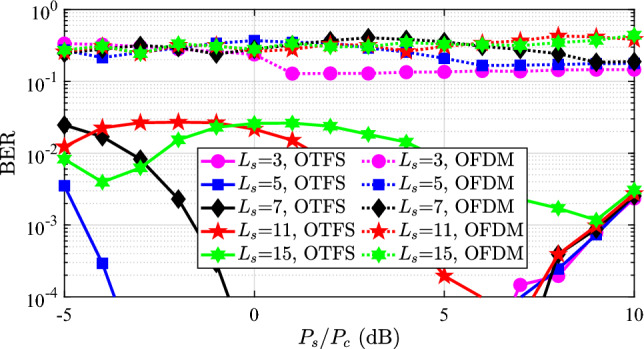
The BER performance with different power allocation $P_s$ and quantization order $L_s$ using the OTFS and OFDM modulation.

As shown in Fig. 2, we first show the bit error rate (BER) performance under different power allocation indices $P_s$ and quantization orders $L_s$. We see that communication performance is good when the quantization order is smaller, such as $L_s = 3, 5, 7$. When the quantization order becomes higher, the BER is worse. Meanwhile, we note that when $P_s$ is much larger than $P_c$, the communication performance is worse than the case where $P_s$ is larger than $P_c$. The reason is the overly small $P_c$ leading to the degradation of communication anti-noise ability. If the total transmitting power $P_t$ is increased, the communication performance will improve. More importantly, we note that when $P_s$ is smaller than $P_c$, the communication performance with a lower quantization order is still good. Generally speaking, this is considered impossible because the sensing interference cannot be properly removed in this case. However, due to the processing gain of OTFS modulation for FMCW signal, the power of the sensing signal is enhanced, resulting in correct demodulation even when $P_s<P_c$. In addition, we also simulate the case where the UAV adopt the OFDM modulation to generate the power-domain ISAC waveform, and the results are plotted in Fig. 2. Obviously, the communication BER is terrible than the case using OTFS modulation because the frequency shift caused by the high-speed moving can not be handled well. The results highlight the advantage of the OTFS modulation in the high-speed moving scenario.

**Fig. 3:**
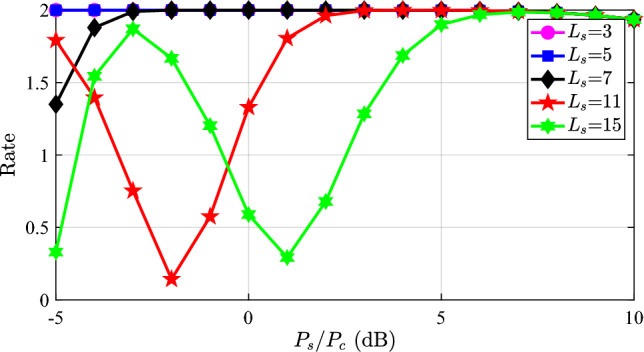
The communication rate with different power allocation $P_s$ and quantization order $L_s$ using the OTFS modulation.

Moreover, we also show the communication rate in Fig. 3. From Fig. 3, we see that the communication rate is almost $\log _2 M_c$ for a smaller quantizer at any $P_s/P_c$, which means the communication information is successfully conveyed. Similarly, with the increase of the quantization order $L_s$, the rates also decrease. The varying rate curves correspond to the BER curves in Fig. 2. The reason for the variation is that the power ratio $P_s/P_c$ does not linearly impact the communication performance. When $P_s/P_c$ increases initially, it means the power of the communication signal is improved, while the power of the sensing signal is much larger than that of the communication signal. Therefore, the communication signal cannot interfere with the sensing signal, leading to the successful demodulation of the sensing symbol. Subsequently, the communication symbols are demodulated without sensing interference, and the performance is enhanced with the communication power. However, when the $P_s/P_c$ increases further, the communication power is basically the same as the sensing power, resulting in the failure of demodulating the sensing signal. The residual sensing signal interferes with the communication signal, degrading the communication performance.

### Sensing performance

**Fig. 4:**
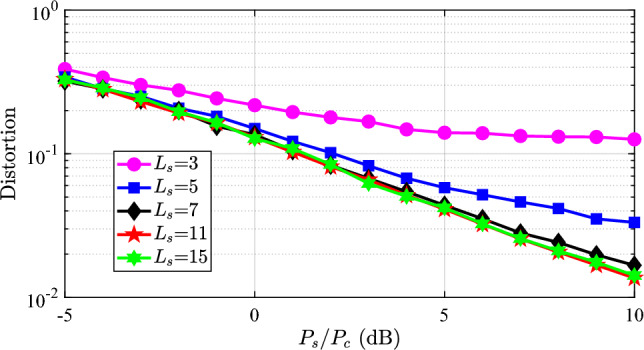
The sensing distortion with different power allocation $P_s$ and quantization order $L_s$ using the OTFS modulation.

Then, we discuss the distortion metric of the sensing function in Fig. 4. The variation of the distortion is simple compared with the communication performance. The distortion always decreases as the $P_s/P_c$ and $L_s$ increase. The reason is evident: the slighter the quantization, the more components in the ISAC waveform, and the smaller distortion is obtained. The distortion performance is not impacted by the additive noise. Additionally, we should note that with higher quantization orders, such as $L_s = 7, 11,$ and 15, the distortion performances are almost the same. This result means we could choose a lower quantization order to guarantee the communication performance while keeping the distortion almost invariant. An overly high quantization order not only brings little sensing performance gain but also severely degrades the communication performance.

**Fig. 5:**
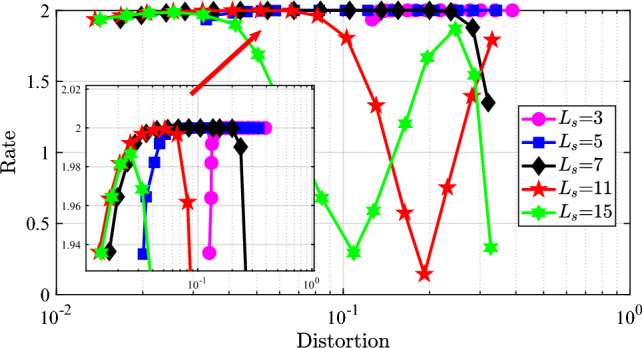
The rate-distortion trade-off performance of the proposed waveform.

### Trade-off performance

For the ISAC systems, the most important thing is that the system should have flexible controllable performance between sensing and communication functions. In order to show the flexibility, we plot the rate-distortion performance of the proposed waveform in Fig. 5. In Fig. 5, we see that the quantization has the obvious influence on the balance between sensing and communication performance. When the quantization order is small, i.e., $L_s = 3$, the sensing distortion is much larger than other cases of quantization order, which means the small quantization order can not achieve a good trade-off performance. With the quantization order increasing, the curves are moving to the left-up corner, where the optimal trade-off performance occurs. Especially, when the quantization order $L_s = 11$, the optimal trade-off is achieved, as shown in the mini-figure in Fig. 5. These results show that the adjustable parameters $L_s$ and $P_s$ could satisfy the requirement of the UAV-ISAC systems.

## Conclusions

Integrated sensing and communication systems play a crucial role in advancing radio communication and sensing in the sixth-generation era. Simultaneously, unmanned aerial vehicles enhance communication and sensing capabilities by offering expansive coverage and efficiency, thanks to their high mobility. Combining these technologies holds great promise for the future of wireless communication. However, a key challenge lies in devising a suitable transmitting waveform that caters to high-mobility UAV communication while maintaining robust sensing capabilities. In this study, we advocate for integrating OTFS with FMCW using power-domain superposition to enable efficient high-mobility communication and sensing. OTFS modulation addresses Doppler shifts in communication, while FMCW waveform ensures effective sensing. We also explore the trade-off between sensing and communication functions concerning ratio distortion. Numerical results affirm the efficacy of our proposed waveform design.

The datasets generated during and/or analysed during the current study are available from the corresponding author on reasonable request.

## Data Availability

The datasets generated during and/or analysed during the current study are available from the corresponding author on reasonable request.
